# Teaching clinical skills in the theatre of medicine

**DOI:** 10.1007/s40037-018-0445-6

**Published:** 2018-07-27

**Authors:** Gerard J. Gormley, Paul Murphy

**Affiliations:** 1 Clinical Skills Education Centre, Centre for Medical Education, Queen’s University Belfast, Belfast, UK; 2 Brian Friel Centre, School of Arts, English and Languages, Queen’s University Belfast, Belfast, UK

## Introduction

Many doctors will fondly look back on their medical student days. Aside from grappling with theoretical knowledge, ‘hands-on’ experience was eagerly anticipated as they embarked on their journey as being doctors of the future. This was no more evident than in the teaching of physical examination skills, using their senses to deduct key observations from a patient and thereby establishing a diagnosis. Whether the ‘squeaky’ noise (like walking in fresh laid snow) of a pleural rub, observing the ‘pill-rolling’ tremor of Parkinson’s disease or feeling the ‘olive’ like consistency of a swollen lymph node, making sense of these observations is foundational in doctor training. Such abstract phenomena were and remain challenging for junior medical students to conceptualize. Inspirational teachers could, however, convey such complexities with ease. The image of my first clinical teacher spontaneously taking a group of students to percuss the side of a water fountain tank—to audibly establish the air/fluid level—as in patients with pleural effusion (i. e. a condition where there is a collection of fluid in the space around the lungs) remains as clear and remarkable to me as the day it happened. As eloquently described by our teacher, the physical effect of the lesson was analogous with the wine makers of old who percussed wine casks to see how much wine was left. Not only are such memories and sense-making long lasting, but also the impression of the expert who so effortlessly taught us. Such improvisational teaching techniques were, looking back, powerful methods that assisted our sense-making. In Harris and Rethans’ study they have masterfully explored the use of creativity and improvisation in the teaching of physical examination skills by unearthing the importance of such creative skills that all too often are below surface awareness [1]. Yet their findings will no doubt resonate with clinicians, students and teachers alike.

Whilst taking a medical history from a patient largely involves the use of language to elicit key clinical information, physical examination uses a range of sensory channels to detect relevant clinical findings. As highlighted by Harris and Rethans, physical examination skills often require more imaginative, embodied experiences for learners to conceptualize [1]. Language alone can so often fall short of painting the entire picture of a physical sign, especially when the same clinical sign can vary from patient to patient and even from day to day. Whilst textbooks can lay down the theoretical aspects of physical examination skills and signs, they can only take learners to a point of understanding. Creative teaching techniques bring knowledge from the textbook into a more embodied, tacit experience for learners.

## Learning from the dramatic arts

Harris and Rethans make the salient analogy with the improvisation of a jazz pianist, noting the difficulty of the task and the dedication and training required to achieve a high calibre of improvisation [1]. In the field of dramatic art Konstantin Stanislavski, the doyen of modern actor training methods, highlighted the importance of improvisation in achieving high-quality performance [2]. Stanislavski distinguished between on the one hand ‘instinctive’ actors who rely solely on inspiration and see ‘no need for technique at all’ and on the other hand those who had developed their technical skills to a level where their improvisation could bring ‘freshness and spontaneity’ to the role they were playing [2].

Trained actors could improvise in terms of manifold aspects of their performance and particularly during rehearsal as they developed their role and the sequence of actions that would define their character’s journey through the play. Actors could improvise on an imaginary theme, for example having a stranger with a threatening intent—appear at the door of a mundane, domestic living room scene, that would force the actors to alter the contours of their character’s emotional state. Actors could improvise in terms of their physicality, for example performing different ways of carrying a tray across a room, either elegantly or as if they were intoxicated or injured, in order to alter the ‘tempo-rhythm’ of their performance [2]. Actors could similarly practice alternating between ‘arrhythmic action and rhythmic speech’ and ‘rhythmic action and arrhythmic speech’ to further nuance their role. The parallel with medical education in this context then is to expand the common understanding of skills, specifically to compliment clinical skills with behavioural skills, both of which of course can use improvisation but when considered in a holistic, systemic approach can add a multiplicative rather than additional value to the learning experience.

In ‘The Presentation of Self in Everyday Life’, Erving Goffman used theatre as an interesting metaphor to explain the dynamics of social interaction [3]. In his dramaturgical metaphor, he described individuals as actors in a play who put on a show for others. He drew similarities between stage acting and the performance of professional roles. In the *theatre* of medical education, the *teacher* is the *main actor* and *students* the *audience*. Instead of entertainment, the principal role of teachers is to educate and have a transformative impact on learners. Like any professional actor, long hours of practice and training are required to produce a professional performance. So too are educators in the *theatre* of teaching. Novice teachers can read a *script* (clinical textbook content), but expert teachers deliver a *script* with sincerity that engages an audience and captures their imagination. There are many methods of acting and, as in drama, there are also many methods of teaching in education.

As Stanislavski noted, improvisation in acting requires technical training as the innate act is not sufficient in and of itself [2]. In this sense, improvisation is a key aspect in what Stanislavski called ‘the method of physical actions’ which he developed to enhance the fidelity of an actor’s performance in their aim of winning over an audience and achieving acclaim. So too in medical education where a skilled educator requires proper training and guidance. Creative techniques can provide a potent method to achieve the aim of enhancing student learning. Such creative techniques have been used for many centuries, even back to the times of Hippocrates. What Harris and Rethans have brought to the evidence base are deep insights into these often overlooked methods of teaching [1]. Such insights have promise in guiding the training of clinical educators in their teaching of clinical skills. As they alluded to in their article, a recipe book can instruct how to make a dish, but an expert teacher can provide room for creativeness and experimentation to produce a Michelin star meal.
